# Immediate effects of spinal manipulation on thermal pain sensitivity: an experimental study

**DOI:** 10.1186/1471-2474-7-68

**Published:** 2006-08-15

**Authors:** Steven Z George, Mark D Bishop, Joel E Bialosky, Giorgio Zeppieri, Michael E Robinson

**Affiliations:** 1 Department of Physical Therapy, Brooks Center for Rehabilitation Studies, PO Box 100154, University of Florida, Gainesville, FL, 32610-0154, USA; 2 Department of Physical Therapy, PO Box 100154, University of Florida Gainesville, FL 32610-0154, USA; 3 Department of Physical Therapy, PO Box 100154, University of Florida Gainesville, FL 32610-0154, USA; 4 SHANDs and the University of Florida, PO Box 100154, Gainesville, FL 32610-0154, USA; 5 Department of Clinical and Health Psychology, Center for Pain Research and Behavioral Health, University of Florida, Gainesville, FL 32610-0165, USA

## Abstract

**Background:**

The underlying causes of spinal manipulation hypoalgesia are largely unknown. The beneficial clinical effects were originally theorized to be due to biomechanical changes, but recent research has suggested spinal manipulation may have a direct neurophysiological effect on pain perception through dorsal horn inhibition. This study added to this literature by investigating whether spinal manipulation hypoalgesia was: a) local to anatomical areas innervated by the lumbar spine; b) correlated with psychological variables; c) greater than hypoalgesia from physical activity; and d) different for A-delta and C-fiber mediated pain perception.

**Methods:**

Asymptomatic subjects (n = 60) completed baseline psychological questionnaires and underwent thermal quantitative sensory testing for A-delta and C-fiber mediated pain perception. Subjects were then randomized to ride a stationary bicycle, perform lumbar extension exercise, or receive spinal manipulation. Quantitative sensory testing was repeated 5 minutes after the intervention period. Data were analyzed with repeated measures ANOVA and post-hoc testing was performed with Bonferroni correction, as appropriate.

**Results:**

Subjects in the three intervention groups did not differ on baseline characteristics. Hypoalgesia from spinal manipulation was observed in lumbar innervated areas, but not control (cervical innervated) areas. Hypoalgesic response was not strongly correlated with psychological variables. Spinal manipulation hypoalgesia for A-delta fiber mediated pain perception did not differ from stationary bicycle and lumbar extension (p > 0.05). Spinal manipulation hypoalgesia for C-fiber mediated pain perception was greater than stationary bicycle riding (p = 0.040), but not for lumbar extension (p = 0.105).

**Conclusion:**

Local dorsal horn mediated inhibition of C-fiber input is a potential hypoalgesic mechanism of spinal manipulation for asymptomatic subjects, but further study is required to replicate this finding in subjects with low back pain.

## Background

There is considerable evidence suggesting that spinal manipulative therapy (SMT) is an effective treatment for subgroups of patients with low back pain (LBP) [1-4]. Furthermore, a clinical prediction rule indicative of optimal treatment outcomes from SMT has been proposed [5] and validated [6]. While the clinical literature provides strong support for the use of SMT for certain patients, its underlying effects and mechanisms are not widely understood.

SMT is frequently theorized to correct mechanical lesions, such as "subluxation" or "segmental dysfunction", despite the lack of empirical support for such theories [7,8]. SMT does appear to have demonstrable mechanical (i.e. peak forces and displacement) effects on spinal segments [9-17]. However, there is skepticism as to whether therapeutic benefits of SMT can be solely attributed to these effects. First, peak forces generated from SMT vary greatly by practitioner, suggesting this factor is not related to clinical improvement [18,19]. Second, lasting positional changes following SMT have not been observed [20], suggesting this factor is also not related to clinical improvement.

Neurophysiological processes have also been used to explain the underlying effect of SMT [13,21-23]. Specific to the purposes of this study, SMT has been theorized to affect spinal joint and muscle spindle mechanoreceptors, activating low (A-beta) and high (A-delta, C) threshold afferents [7]. This afferent input converges on the spinal cord with the potential to inhibit dorsal horn cells involved with transmission or amplification of nociceptive input. In this scenario, SMT's underlying effect would be as a "counter-irritant" stimulus to peripheral nociceptive input received by dorsal horn cells [7].

If these neurophysiological processes occurred, SMT would have a measurable hypoalgesic effect on pain perception. This topic was reviewed by Vernon [24], with SMT hypoalgesia observed by decreased cutaneous receptive field from pin-prick [25], tolerance from electrical current [26], and mechanical pressure [27], Collectively, these results demonstrate SMT's potential for dorsal horn mediated pain inhibition.

There are, however, several important, unresolved issues regarding SMT hypoalgesia. The previously cited studies investigated hypoalgesia in anatomical areas with the same or overlapping dermatomes as those affected by SMT [25-27], For example, assessing hypoalgesic response to cervical manipulation only in anatomical areas innervated by cervical nerve roots [27]. As a result, these studies were unable to determine if the observed hypoalgesia was a large, general effect or a specific effect local to the spinal levels involved with the manipulation [24]. Previous studies utilized pain induction protocols assessing general peripheral pain perception, instead of distinguishing SMT's separate effect on A-delta and C-fiber mediated pain perception [28]. Last, the previously cited literature included comparisons with a true control group [26], detuned short-wave therapy [25], and oscillatory mobilization [27]. Subjects performing general physical activity or specific back exercises are clinically relevant comparison groups missing from the current SMT hypoalgesia literature.

Consequently, the present study investigated the immediate hypoalgesic effects of lumbar SMT on thermal pain sensitivity in asymptomatic subjects. We selected thermal stimuli for pain induction because unlike other experimental pain methods, it offered the sensitivity to test different anatomical areas and separate testing of A-delta fiber and C-fiber mediated pain perception [28-30]. Our first purpose was to determine if lumbar SMT hypoalgesia was a locally observed phenomenon by demonstrating a) hypoalgesia in lumbar innervated sites, but not in the control (cervical innervated) sites and b) hypoalgesia had a low correlation with relevant pain-related cognitions. Our second purpose was to determine if lumbar SMT hypoalgesic effects were a) greater than hypoalgesia from physical activity and b) different for A-delta fiber or C-fiber mediated pain responses.

## Methods

### Subjects

This sample was comprised of undergraduate and graduate students who responded to study advertisements placed in health science classrooms of a large research university. Subjects read and signed a consent form that had been approved by University's Institutional Review Board before participating in any study-related procedures. Subjects were verbally screened for history of LBP and current use of pain relievers. Subjects not currently experiencing LBP and not using pain relievers were included in this study.

### Procedure

Demographic information, previous pain experience, psychological questionnaires, and thermal pain sensitivity measures were collected before intervention was randomly assigned. Each of the randomly assigned interventions was applied for a standard 5-minute period to minimize variation in hypoalgesic effect due to differences related to re-assessment time and treatment dosage. The thermal pain sensitivity measures were collected again 5 minutes after intervention was administered. Our rationale for only measuring immediate effects was twofold. First, this was a preliminary study and we wanted to confirm that we could detect hypoalgesic effects on thermal sensitivity under ideal circumstances. Second, this study involved asymptomatic subjects and we did not expect them to have a long-term response to these interventions because of a lack of appropriate disease process.

### Measures

#### Psychological questionnaires

We selected psychological variables with previously reported influence on quantitative sensory testing [31-34].

The *Fear of Pain Questionnaire *(FPQ-III) uses a 30-item, 5-point rating scale to measure fear about specific situations that would normally produce pain [35]. The FPQ-III is a commonly used and well-validated instrument that is appropriate for use in non-clinical and clinical populations [35-37].

The *Coping Strategies Questionnaire *(CSQ) uses a 27-item, 7-point rating scale to measures the frequency of use for common pain coping strategies [38]. The CSQ is commonly used in pain studies and is appropriate for use in non-clinical and clinical populations. We utilized the catastrophizing subscale that measures helplessness and pessimistic cognitions related to pain perception. The validity of this particular subscale has been supported [38-41] and the currently recommended scoring system was used in this study [40].

The *State-Trait Anxiety Questionnaire *(STAI) uses a 40-item, 4-point rating scale to assess dispositional (trait) and situational (state) anxiety symptoms [42]. The STAI is commonly used to assess anxiety and is appropriate for use in non-clinical and clinical populations. We reported the state portion of the STAI as this construct better matched the purposes of this study.

The *Anxiety Sensitivity Index (ASI) *uses a 16-item, 4-point rating scale to assess anxiety sensitivity, which is the perception of whether experiencing symptoms of anxiety causes harm. The ASI is commonly used in pain studies and is appropriate for use in non-clinical and clinical populations. The ASI has been validated in community samples [43] and has demonstrated factor invariance across different sex and age groups [44].

#### Thermal pain sensitivity

Subjects underwent quantitative sensory testing as per previously established protocols involving thermal stimuli [29,30,45,46]. We selected this protocol because unlike other methods of experimental pain induction thermal stimuli is sensitive to A-delta fiber and C-fiber mediated pain perception. We used a protocol with parameters that parallel those from basic studies [28] and was successful in detecting hypoalgesic response for healthy controls taking fentanyl [29].

Thermal stimuli were delivered via contact thermode and a computer-controlled Medoc Neurosensory Analyzer (TSA-2001, Ramat Yishai, Israel) with a hand-held, peltier-element-based stimulator. In our pilot testing of this protocol (n = 10), we included a stimulation site involving lumbar paraspinal musculature. However, subjects were unable to distinguish between A-delta fiber and C-fiber mediated pain perception, in comparison to testing in the extremities. We attributed this difference to the relatively short distance the thermal stimuli had to travel to the dorsal horn from the lumbar musculature. This short distance did not allow subjects to differentiate input based on fiber type. Therefore, we limited pain perception testing to extremity areas innervated by lumbar and cervical dermatomes in the present study.

Stimuli were applied to the subjects' non-dominant sides and stimulus sites included areas innervated by lumbar dermatomes (the plantar surface of the foot and the posterior calf). Control sites included areas innervated by cervical dermatomes (the volar surface of the hand and forearm). Order of stimulation sites was counter-balanced to prevent ordering effects and exact stimulation sites were varied to prevent carryover effects due to spatial summation, local sensitization, or suppression of nociceptors. The interval between stimuli was at least 60 seconds to avoid carryover effects for the preceding thermal stimulus. Subject response to thermal stimuli was determined with a numerical rating scale (NRS) for evoked pain intensity. The NRS for evoked pain intensity ranged from "0" (No pain) to "100" (Worst pain intensity imaginable).

Subjects were familiarized to the thermal stimuli with a practice session. In the practice session, a continuous heat stimulus was delivered to the subjects' dominant arm. The stimulus started at 35°C and was increased at a rate of 0.5°C with subjects terminating the stimulus when the temperature reached pain threshold. This was repeated three times and the average threshold was calculated. In addition to familiarizing the subjects to thermal stimuli, the pain threshold data allowed us to investigate if the intervention groups were confounded by general pain sensitivity. We then assessed specific components of thermal pain sensitivity from previously reported protocols [29,30,45].

#### First pain response

Heat stimuli of 3 seconds duration were applied to the subjects' skin. The temperature rose rapidly (10°C/sec) from a baseline of 35°C to a randomly determined peak of 45, 47, 49, or 50°C. The research assistant recorded NRS ratings of pain intensity. Subjects were asked to rate their "first" pain intensity felt. These ratings are believed to be primarily mediated by input from A-delta fibers [28,29].

#### Temporal summation

A train of 10 consecutive heat pulses of <1 second duration at an inter-stimulus interval of .33 Hz was delivered to the subjects. A frequency of .33 Hz was selected to ensure the development of temporal summation [28]. The temperature of the heat pulses rapidly fluctuated (10°C/sec) from a low of 35°C to a peak of 47°C. Temperature levels were monitored by a contactor-contained thermistor, and returned to a preset baseline of 35°C by active cooling. The research assistant recorded NRS ratings of pain intensity. Subjects were asked to rate their delayed (second) pain intensity associated with the first, third, and fifth heat pulses. These ratings are believed to be primarily mediated by C-fiber input [28,29].

### Intervention

Subjects were given a standard instructional set that each intervention was commonly used as part of LBP treatment. Subjects were then randomly assigned to receive one of the following interventions. All interventions were performed under the supervision of research staff to ensure compliance with the described parameters.

Stationary bicycle. Subjects rode a stationary bicycle for 5 minutes at 60–70 rpm and 1 KP. This intervention served as a non-specific, active comparison group with which to compare specific active and passive interventions used to treat spine pain. Our rationale for not including a control group is that we wanted the comparison group for this study to account for non-specific effects related to performing general physical activity.

Lumbar extension exercise subjects performed a prone extension exercise previously described in the literature for treatment of LBP [47,48]. This exercise involves the patient lying flat in a prone position. Then, the patient used his/her arms to press his/her chest of the treatment table, and extending the lumbar spine. Subjects were given verbal cues to maintain their hips in contact with the treatment table to prevent substitution from other anatomical areas. Several studies support the effectiveness of this exercise and no adverse events have been reported [49-53]. Subjects performed 3 sets of 15 repetitions within a 5-minute period.

**Figure 1:**
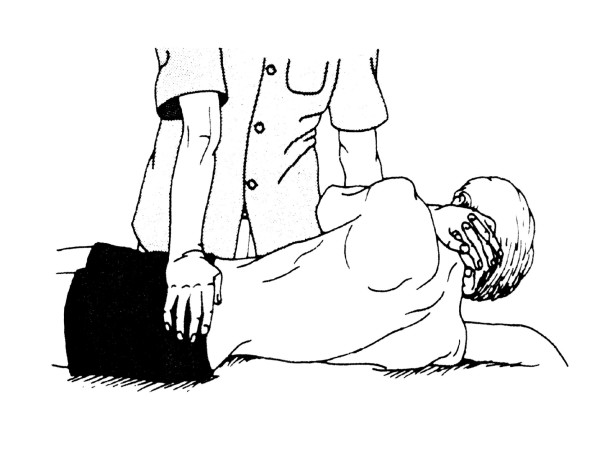
**Spinal manipulation technique utilized in this study(5;47;54–56)**. (Reprinted with permission of the American Physical Therapy Association from Cibulka MT. The treatment of the sacroiliac joint component to low back pain: a case report. *Phys Ther*. 1992;72:917–922.)

SMT. Subjects received a lumbar SMT previously described in the literature for treatment of LBP (Figure 1) [47]. This SMT technique is performed with the patient supine, and the researcher standing on the opposite side of the table. The researcher passively side bent the patient toward the side to be manipulated and asked the subject to interlock hands behind his/her head. The researcher then passively rotated the subject away from the side to be manipulated and delivered a posterior and inferior thrust to the opposite ASIS. Several randomized trials support the efficacy of this specific technique and no adverse events have been reported [5,54-56]. The SMT was performed four times within a 5-minute period, alternating thrusts between right and left ASIS's. Specifically, the researcher applying the manipulation was trained to pace the repositioning process to take 1 minute, allowing 10–15 seconds to perform the thrust.

### Data analysis

All data analyses were performed using SPSS for Windows (SPSS Inc, 233 S. Wacker Drive, 11th floor, Chicago, Illinois 60606), Version 13.0 at a Type I error rate of 0.05. Descriptive statistics were generated for the demographic, psychological, and pain threshold measures. Randomization effect was investigated by comparing treatment groups with one-way ANOVA. Any observed group differences were considered as covariates in the subsequent analyses.

Our purposes were investigated by testing for group × time interactions for either first pain response or temporal summation in the lumbar and cervical innervated testing sites. First pain response for 47 and 49°C was tested with repeated measures ANOVA. Data for 45 and 50°C were not presented because these data represented sub-threshold (i.e. floor effect for hypoalgesia) and tolerance (i.e. ceiling effect for hypoalgesia) values for a majority of patients, respectively. Treatment group [3] and pre and post NRS first pain ratings [2] were the model factors. Temporal summation was tested with repeated measures ANOVA, with treatment group [3] and pre and post NRS temporal summation ratings [2] as the model factors. The 3 primary analyses involving repeated measures ANOVA models were performed without correction of the Type I error rate. This strategy was selected because this was a preliminary study and we wanted to utilize a liberal definition of statistical significance to avoid potential misinterpretation of the data. However, any post-hoc testing was performed with Bonferroni correction of the 0.05 Type I error rate. We also calculated Pearson product correlations between psychological variables, pain threshold measures, and SMT hypoalgesia.

## Results

The 3 treatment groups did not significantly differ on the demographic, previous pain experience, psychological, and pain threshold measures (Table 1). There were no significant group × time interactions for first pain hypoalgesia in the cervical innervated sites at either 47°C (F_1,57 _= 0.4, partial η^2 ^= 0.02, p = 0.645) or at 49°C (F_1,57 _= 0.3, partial η^2 ^= 0.01, p = 0.720). In addition, there was no significant hypoalgesia (i.e. treatment effect) for the cervical innervated sites at either temperature (Table 2). Similarly, there was no significant group × time interaction for temporal summation hypoalgesia in the cervical innervated sites (F_1,57 _= 0.5, partial η^2 ^= 0.02, p = 0.620) and there was no general temporal summation hypoalgesic effect in the cervical innervated sites (F_1,57 _= 0.7, p = 0.405).

**Figure 2:**
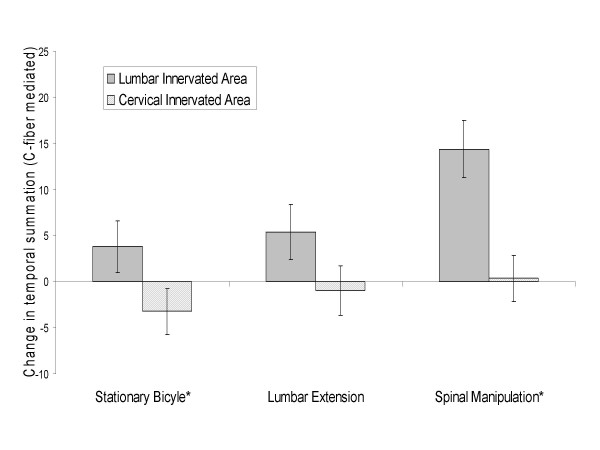
**Temporal summation hypoalgesia for stationary bicycle, lumbar extension, and spinal manipulation**. Figure 2 Key • Positive numbers indicate hypoalgesia • Error bars are 1 standard error • * – indicates statistically significant (p < 0.05) difference in intervention for pain sensitivity in lower extremity area.

There were no significant group × time interactions for first pain response hypoalgesia in lumbar innervated sites at either 47°C (F_1,57 _= 2.4, partial η^2 ^= 0.08, p = 0.101) or at 49°C (F_1,57 _= 1.3, partial η^2 ^= 0.05, p = 0.268). However, there was a significant hypoalgesia (i.e. treatment effect) on the lumbar innervated sites at both temperatures. All interventions were associated with first pain hypoalgesia, but only SMT had a consistent association (Table 2). There was a significant group × time interaction for temporal summation hypoalgesia in the lumbar innervated sites (F_1,57 _= 3.7, partial η^2 ^= 0.12, p = 0.030). Temporal summation hypoalgesic responses are included in Figure 2 to allow descriptive comparisons of the cervical and lumbar innervated responses. Post-hoc testing revealed that SMT had a larger hypoalgesic effect in the lumbar innervated sites than stationary bicycle (p = 0.040), but similar as lumbar extension exercise (p = 0.105).

The Pearson correlations among the psychological, pain threshold, and SMT hypoalgesic response variables were generally low, ranging from -0.31 to 0.25, and none reached statistical significance (Table 3).

## Discussion

This study investigated the immediate hypoalgesic effect of lumbar SMT on thermal pain sensitivity in asymptomatic subjects. The first purpose was to investigate whether SMT hypoalgesia was a local phenomenon. This purpose adds to the existing literature because previous studies have demonstrated SMT hypoalgesia by testing anatomical sites primarily affected by the manipulative technique [25-27]. As a result, there is a question whether SMT hypoalgesia was the result of a general or local nervous system response [24]. Our results support SMT hypoalgesia as primarily a local phenomenon. First, there were no hypoalgesic effects observed in cervical innervated sites, but there were hypoalgesic effects observed in the lumbar innervated sites. The implication of this finding is that the dorsal horn inhibition from SMT did not have a wide-ranging effect on peripheral input received from lumbar and cervical dermatomes. Second, there were no statistically significant or large correlations between pain-related cognitions, pain threshold, and the SMT hypoalgesic response. For example, the largest correlation was with state anxiety (r = -0.31), suggesting this cognition accounted for only 9.6% variance in the hypoalgesic response. The implication of this finding is that psychological influences on SMT hypoalgesic response were likely not present, or only a minor influence.

Our findings suggests SMT hypoalgesia is potentially a local neurophysiological phenomenon in asymptomatic subjects, corroborating with other studies demonstrating local SMT effects for EMG activity [57,58] and inflammation control [59]. However it must also be considered that the literature supports the potential of a central mechanism for SMT hypoalgesia. Specifically, it has been proposed that SMT hypoalgesia is a result of the activation of endogenous descending pain inhibitory systems mediated through the periaqueductal gray region of the midbrain [60]. In a human study this central mechanism was supported by Vincenzino et al[61] who reported hypoalgesia from cervical manipulation was significantly correlated (r = 0.82) with sympathoexcitation. In an animal study this central mechanism was supported by Sykba et al [62] who reported hypoalgesia from knee manipulation was not affected by local spinal blockade of GABA or opioid receptors. Therefore, the current literature provides available evidence suggesting SMT hypoalgesia may be resultant of local and/or central mechanisms.

Our second purpose was to investigate whether SMT hypoalgesia differed from physical activity for first pain response or temporal summation. This purpose adds to the existing literature because previous studies of SMT hypoalgesia have not included these clinically relevant comparisons and have not used protocols that differentiated between A-delta and C-fiber mediated pain perception [24]. Our results provided information supporting SMT as a "counter-irritant" to inhibit peripheral noxious stimuli at the dorsal horn [7]. SMT appeared to have a general counter-irritant effect on A-delta fiber mediated pain perception (first pain response). SMT had a consistent hypoalgesic effect on A-delta fiber mediated hypoalgesia, while stationary bicycle riding and lumbar extension exercise hypoalgesic effects were noted only at 47°C. SMT appeared to have a specific counter-irritant effect on C-fiber mediated pain perception (temporal summation), as SMT hypoalgesia was greater than bicycle riding and trended toward being greater than lumbar extension exercise.

The specific hypoalgesic effect on C-fiber mediated pain perception is an intriguing finding and could provide partial explanation for the clinical effectiveness of SMT. Numerous basic studies have suggested that central sensitization of pain is a specific neurophysiological mechanism associated with the development and maintenance of chronic pain syndromes [63-68]. Wind-up results from tonic, peripheral nociceptive C-fiber input and is an example of central sensitization that occurs within dorsal horn cells. This input activates NMDA and substance P receptors in wide dynamic range and nociceptive specific cells. Then, the tonic activation of these cells induces a central hyperalgesia mediated at the spinal cord level, such that subsequent evoked pain stimuli are relayed from the dorsal horn as increasing in intensity, despite their being of standard amplitude. In basic models, this temporal parameter (increasing frequency of nociceptive input) is a primary factor in eliciting wind-up [65].

Direct measurement of wind-up is not feasible in human subjects, but temporal summation of thermal stimuli is an accepted behavioral measure of wind-up [28]. The use of temporal summation as a proxy measure of wind-up is supported by human studies that demonstrate an increase in the frequency of standard nociceptive input increases the report of pain perception [29,30,45]. Specifically, thermal input at .33 Hz or less tends to induce temporal summation in humans, while input at .20 Hz or greater does not [28]. The results of the present study indicated that SMT reduced temporal summation, suggesting a potential underlying effect of SMT is the inhibition of dorsal horn wind-up [7]. Inhibition of dorsal horn wind-up would mean the individual was less likely to develop chronic LBP, at least chronic LBP caused by this particular pain mechanism. It should be noted that this explanation is speculative at this time, as only one study directly links temporal summation with chronic LBP [69]. One interpretation of these data is that SMT has the potential of inhibiting dorsal horn windup from peripheral noxious stimuli. While this an intriguing explanation, we acknowledge that these findings may also be explained by other unrelated factors, such as non-specific effects related to differences in active and passive interventions.

This experimental model offers several advantages in the study of SMT hypoalgesia. The use of thermal stimuli allowed us to precisely control levels of nociceptive input and differentiate this input based on fiber type. The use of asymptomatic subjects eliminated confounding of the hypoalgesic response from clinical conditions and pain medications. However, there are also several limitations to consider when interpreting this study. First, although use of asymptomatic subjects offers advantages, these findings cannot be directly generalized to patients with LBP. In patients with LBP a wider range of psychological scores would be expected, potentially making them more robustly related to hypoalgesia from SMT. Also, patients with LBP experience ongoing, nociceptive input that is likely to result in enhanced temporal summation in comparison to asymptomatic controls, thereby interacting with the proposed mechanisms of SMT. Second, this study only tested the immediate hypoalgesic effects of SMT and utilized standard treatment parameters that did not mimic clinical settings. This methodology was necessary to provide the internal validity to detect a short-term hypoalgesic response, however this methodology also means that no assumptions can be made about longer-term hypoalgesic effects or the effect of these particular interventions applied under different parameters. Third, we did not include sham SMT in this study, so we were unable to account for hypoalgesic effects associated with the specific expectation of SMT being a successful intervention for pain relief in lumbar innervated areas. Fourth, we did not report joint cavitation in this study because previous work demonstrated considerable variability in the location of cavitation for lumbar SMT [70], and experiencing cavitation does not appear to affect EMG activity [13] or pain outcomes [71,72]. Last, although we did implement a comparison group (bicycle), we did not utilize a control group in our current design. This methodological selection means that non-specific effects related to differences in active (bicycle and prone press ups) and passive (SMT) interventions are a viable alternate explanation to our findings.

## Conclusion

The finding that SMT may have a local hypoalgesic effect specific to C-fiber mediated input for asymptomatic subjects adds to the previous literature and provides direction for future study. First, this experiment should be reproduced in patients with LBP that have not yet received other pain treatments. Such studies will provide important information on whether this potential pain inhibiting mechanism is also observed in patient populations. Second, future studies should include longer follow-up times and investigate the dose-response relationships between SMT and hypoalgesia. This topic has been under-reported in the SMT literature [24] and such studies will indicate the duration of SMT hypoalgesia and the minimum dosage to achieve optimal SMT hypoalgesia. Third, future studies should include control groups and techniques that block peripheral nociceptive input at the level of the dorsal horn. Inclusion of such methodology will allow future researchers to confirm or refute our mechanistic interpretation of these findings.

## Abbreviations

SMT – Spinal manipulative therapy

LBP – Low back pain

## Competing interests

The author(s) declare that they have no competing interests.

## Authors' contributions

All authors read, edited, and approved the final version of the manuscript.

SZG was responsible for the initial conception of the research question, securing funding, supervising the protocol, data analysis, and manuscript preparation.

MDB and MER were responsible for modifying the research question and critically reviewing earlier versions of the manuscript.

GZ and JEB were responsible for administering the protocol and critically reviewing earlier versions of the manuscript.

## Pre-publication history

The pre-publication history for this paper can be accessed here:



## Figures and Tables

**Table 1:** Descriptive statistics for sample

**Variable**	**Stationary Bicycle (n = 20)**	**Lumbar Extension (n = 20)**	**Spinal Manipulation (n = 20)**	**p-value**
Age (years)	23.9 (3.4)	24.1 (2.6)	24.1 (3.6)	0.975
Sex (# female, %)	12 (60%)	14 (70%)	14 (70%)	0.741
Worst pain experienced (NRS)	68.9 (18.5)	64.0 (21.8)	59.7 (25.9)	0.436
Fear of pain (FPQ)	82.6 (16.7)	75.1 (13.3)	77.5 (22.6)	0.406
Pain catastrophizing (CSQ-R)	7.6 (3.1)	7.2 (3.7)	7.5 (3.8)	0.955
Anxiety (STAI)	45.3 (10.4)	45.5 (11.6)	45.2 (10.7)	0.996
Anxiety sensitivity (ASI)	19.8 (7.6)	16.0 (7.1)	16.0 (7.2)	0.230
Pain threshold (degrees Celsius)	44.7 (2.4)	45.4 (2.2)	44.8 (2.5)	0.589
Pain threshold rating (NRS)	25.0 (21.0)	28.8 (19.0)	21.3 (15.1)	0.443

Key

All data are reported as mean (standard deviation) ratings, unless otherwise indicated.

NRS = Numerical rating scale

FPQ = Fear of Pain Questionnaire

CSQ-R = Coping Strategies Questionnaire-Revised

STAI = State Trait Anxiety Inventory

ASI = Anxiety Sensitivity Index

**Table 2:** First pain hypoalgesia for stationary bicycle, lumbar extension, and spinal manipulation.

**Variable**	**Stationary Bicycle (n = 20)**	**Lumbar Extension (n = 20)**	**Spinal Manipulation (n = 20)**	**Partial Eta-square#**	**p-value#**
Lumbar Innervated*					
NRS Change @ 47°C	13.2 (17.2)^$^	12.9 (17.9)^$^	23.5 (17.3)^$^	0.08	0.101
NRS Change @ 49°C	1.2 (20.2)	6.3 (22.4)	12.1 (19.7)^$^	0.05	0.268
*Cervical Innervated*^					
NRS Change @ 47°C	-3.0 (13.7)	0.3 (11.6)	0.3 (10.2)	0.02	0.645
NRS Change @ 49°C	1.9 (9.0)	-0.4 (10.1)	1.7 (10.8)	0.01	0.720

Key

NRS = Numerical rating scale

All data are reported as mean (standard deviation) ratings.

Negative numbers indicate increased pain following treatment.

# – Significance and partial eta-square estimate are for the interaction between type of treatment and first pain hypoalgesia

* – Significant overall main effect for lower extremity hypoalgesia at 47°C (F_1,57 _= 53.8, p < 0.001) and at 49°C (F_1,57 _= 5.9, p = 0.018)

^$ ^– Significant within group effect for hypoalgesia (p < 0.05)

^ – No significant main effect for upper extremity hypoalgesia at 47°C (F_1,57 _= 0.4, p = 0.525) and at 49°C (F_1,57 _= 0.6, p = 0.424)

**Table 3:** Associations among pain threshold, psychological variables, and lumbar innervated hypoalgesic responses for subjects receiving spinal manipulation.

	**Temporal Summation**	**First Pain @ 47°C**	**First Pain @ 49°C**
**Fear of Pain (FPQ)**	-0.20 (p = 0.401)	0.01 (p = 0.972)	-0.08 (p = 0.732)
**Pain catastrophizing (CSQ-R)**	-0.08 (p = 0.750)	0.18 (p = 0.442)	0.10 (p = 0.686)
**Anxiety (STAI)**	-0.16 (p = 0.499)	-0.19 (p = 0.422)	-0.31 (p = 0.178)
**Anxiety sensitivity (ASI)**	0.21 (p = 0.370)	0.25 (p = 0.292)	-0.10 (p = 0.674)
**Pain threshold (degrees Celsius)**	-0.26 (p = 0.273)	0.11 (p = 0.656)	0.14 (p = 0.545)
**Pain threshold rating (NRS)**	0.05 (p = 0.843)	-0.19 (p = 0.436)	-0.14 (p = 0.549)

Key

NRS = Numerical rating scale

FPQ = Fear of Pain Questionnaire

CSQ-R = Coping Strategies Questionnaire-Revised

STAI = State Trait Anxiety Inventory

ASI = Anxiety Sensitivity Index
